# A detailed mapping of the food industry in the European single market: similarities and differences in market structure across countries and sectors

**DOI:** 10.1186/s12966-021-01117-8

**Published:** 2021-04-26

**Authors:** Iris Van Dam, Benjamin Wood, Gary Sacks, Olivier Allais, Stefanie Vandevijvere

**Affiliations:** 1 Sciensano, Service of Lifestyle and chronic diseases, Brussels, Belgium; 2 Université Paris-Saclay, INRAE, UR ALISS, 94205 Ivry-sur-Seine, France; 3 Global Obesity Centre (GLOBE), Institute for Health Transformation, Deakin University, Geelong, VIC 3220 Australia

**Keywords:** Food industry, Packaged food, Non-alcoholic beverages, Supermarkets, Quick service restaurants, Europe, Food environments

## Abstract

**Background:**

Food environments are influenced by food industries (packaged food and non-alcoholic beverage manufacturers; supermarkets and quick service restaurants). An important source of this influence is the significant market power held by a limited number of food companies. Market structure analysis, as part of a broader market power research agenda, has received limited attention from the public health community. The aim of this study was to analyse similarities and differences in market structure across countries and industries in the European Single Market.

**Methods:**

The companies with the largest market share at the national level for each industry were identified from Euromonitor sales data in 2017/18. The market structure was assessed by the following metrics: the number of global brand owners with ≥1% market share per country, the number of companies unique for one European Single Market member state, the most sold packaged food and non-alcoholic beverage categories, the number of quick-service restaurants and supermarkets per 1000 inhabitants and market concentration by means of the Herfindahl-Hirschman Index (HHI) and the four firm concentration ratio (CR4). CR4-values > 40% and HHI-values > 2000 indicate concentrated markets with limited competition.

**Results:**

The leading packaged food and non-alcoholic beverage manufacturers and the most sold food and beverage product categories were similar across countries in Europe. The observed levels of concentration were however different. Average CR4-values ranged from 21 to 72% among packaged food product markets and 60 to 76% for non-alcoholic beverage product markets. Average CR4-values for quick service restaurants and supermarkets were 50 and 60%, respectively. Across European countries the same leading quick-service restaurants were identified, while this was not the case for supermarkets.

**Conclusions:**

This study forms an important basis to understand key aspects of market structure of the European food industry, observing clear differences between food industries and European Single Market member states. This has potential implications for the implementation of food environment policies at different levels of jurisdiction.

## Background

Since the second world war, diets and lifestyles in Europe have significantly changed together with the development of the European Single Market (ESM) [1]. In 2016, on average, 59% of the European adult population was classified as being overweight (Body Mass Index, BMI ≥ 25 kg/m^2^) [2, 3]. Overweight is often seen as an issue of individual responsibility, but there are important determinants, such as those related to food environments, that are beyond the control of the individual [4–8].

Food environments are generally defined as: “The collective physical, economic, policy and sociocultural surroundings, opportunities and conditions that influence people’s food and beverage choices and nutritional status” [9]. In many areas around the world, current food environments can be described as environments that make the less healthy food choices the easiest choices, as less healthy foods are often more available, heavily marketed and cheaper [10]. Food companies, including food and beverage manufacturers, supermarkets and quick service restaurants, are considered to play a substantial role in shaping food environments [9, 11, 12]. Food companies directly influence food environments by manufacturing, distributing and marketing food products that are made available to consumers. Food companies also indirectly influence food environments, such as through the deployment of political strategies that serve to shape and influence public opinion and political decision making [12–14]. An important source of this influence – both direct and indirect - on food environments is the significant market power held by a limited number of food companies [12, 13, 15]. Substantial market power can confer dominant food companies with the ability to structure food retail environments and food supply chains to suit their own private interests, and can also allow for the generation of considerable profits above what would be possible in a competitive market environment. These profits can then be used to fund practices that undermine public health (e.g. lobbying, intense marketing) [16, 17].

An important step in examining market power is to analyse the market structure in which firms operate [18]. Although market structure analysis alone does not provide a complete picture of the extent of market power held by firms, it is nevertheless useful in understanding the structural power of firms relative to other market-based actors. Market concentration, in particular, is an informative market structure metric, which, for decades, has been considered a key component of market structure analysis [19]. As market concentration increases, the level of competition in the market generally decreases. In turn, given the inverse relationship between competition and market power, a decrease in the level of competition in a market is generally considered to increase the market power of incumbent companies [18, 20]. However, market structure analysis has not received much attention by the public health community.

This study sets out to analyse similarities and differences in market structure across countries and industries (i.e. packaged food and non-alcoholic beverage manufacturers, supermarkets and quick service restaurants) in the European Single Market (ESM). Following metrics were used: the number of food companies with ≥1% market share per country, the number of companies unique for one ESM member state, the most sold packaged food and non-alcoholic beverage categories, the number of quick-service restaurant and supermarket outlets per 1000 inhabitants; and market concentration measured by the Herfindahl-Hirschman Index (HHI) and the four firm concentration ratio (CR4) [18, 21]. Potential implications of the similarities and differences in market structure across countries and industries for the implementation of policies to improve the food environment at national and European level are discussed.

## Methodology

### Selection of countries

Sales and market share data from all countries within the European Single Market (European Union’s 28 member states and 4 EFTA – European Free Trade Association – members, ESM) were included. The Euromonitor International Passport Global Market Information Database was found to have the best available data for the majority of the selected countries and product markets. Euromonitor is the world’s leading independent provider of strategic market research and collects volume sales data from various sources including trade associations, industry bodies, company financial reports, and official government statistics. These data are validated by food industry representatives.

For this study, data were obtained at the most fine-grained level (212 food subgroups in total) over the period 2009–2018 for packaged food and non-alcohol beverage manufacturers and supermarkets and over the period 2008–2017 for quick service restaurants [21]. For the following member states no Euromonitor data were available: Cyprus, Iceland, Liechtenstein, Luxembourg and Malta. As a result, a total of 27 EU countries were included in this study to represent the ESM, 14 in Western Europe (52%) and 13 in Eastern Europe (48%). For these 27 countries Euromonitor data for both packaged food and non-alcoholic beverage manufacturers and supermarkets were available. For quick-service restaurants, data were only available for 22 out of these 27 member states, of which eight (36%) were in Eastern Europe. Thus, for analyses related to quick service restaurants, Croatia, Estonia, Latvia, Lithuania and Slovenia were excluded.

### Selection of food companies

To obtain a comprehensive overview of the food industry within the ESM, packaged food manufacturers, non-alcoholic beverage manufacturers, quick service restaurants and supermarkets were included in the analysis. Supermarkets were considered both as food and beverage manufacturers, through own-brand products placed on the market, as well as retailers. All food companies with ≥1% market share in at least one of the ESM member states were included. For each food industry, the company with the largest market share at the country level (hereinafter referred to as the leading company), as determined by Euromonitor sales and market share data, was identified. Country-level data on actual (USD) and percent retail sales values were sourced for both the national brand owners and the global brand owners. Throughout the article national brand owners were considered as those companies that have the rights to produce or distribute brands within a country (own brands or through licensing agreements) while global brand owners were considered as the ultimate brand owners, as defined by Euromonitor [22].

For quick service restaurants the Euromonitor category ‘Chained Consumer Foodservice’ and for supermarkets the Euromonitor category ‘Grocery Retailers’ was used. The average number of companies included per industry is presented in Table 1.

**Table 1:** Average number of national and global brand owners with ≥1% market share (MS) included per food industry across countries within the European Single Market. Euromonitor data 2017/18

Food industry	Average number of global brand owners with ≥ 1% MS per country (min – max)	Average number of national brand owners with ≥ 1% MS per country (min – max)
**Packaged food**	14 (7–20)	18 (9–25)
**Non-alcoholic beverages**	13 (9–20)	15 (10–20)
**Quick Service restaurants** ^**a**^	20 (11–27)	18 (14–25)
**Supermarkets** ^**b**^	9 (5–18)	10 (5–19)

^a^*‘Chained Consumer Foodservice’: “Chained units are defined by 10 or more units. An exception is made for international chains that have a presence of fewer than 10 units in a country. In this case, they are still considered to be chained units.” As defined by Euromonitor*

^b^*‘Grocery Retailers’: “Retailers selling predominantly food/beverages/tobacco and other everyday groceries. This is the aggregation of hypermarkets, supermarkets, discounters, convenience stores, independent small grocers, chained forecourt retailers, independent forecourt retailers, food/drink/tobacco specialists and other grocery retailers.” As defined by Euromonitor*

### Data analysis

Analyses were conducted separately for the four food industries using SAS 9.4 (Cary, USA, 2018). At time of data collection, in 2019, the latest available Euromonitor data were used, namely 2018 for packaged food manufacturers, non-alcohol beverage manufacturers and supermarkets and 2017 for quick service restaurants. Earlier data were used to observe changes over time, where relevant. An overview of all metrics used to assess aspects of market structure and their respective interpretation can be found in Table 2.

**Table 2:** Overview of the different metrics used to assess aspects of the market structure and their respective interpretation. ESM = European Single Market

Metrics	Calculation ***(using Euromonitor sales and market share data)***	Interpretation
**Market similarities and differences**		
Leading global brand owner per country	Global brand owner market share data per country	The different (or similar) leading global brand owners across Europe
Leading national brand owner per country	National brand owner market share data per country	The different (or similar) leading companies across Europe that nationally have the right to produce or distribute brands
Number of global brand owners with ≥1% market share per country	The number of all global brand owners per country with ≥1% market share	The higher the number of global brand owners with ≥1% market share the more diverse the food industry was assumed to be
Number of unique companies per country	The number of all companies in a country having presence in only one ESM member state	The higher the number of unique companies, the more diverse the actors active within the food industry were assumed to be
Leading European global brand owners	Sum of the sales data per member state by year and global brand owner	The leading companies that own the most sold brands across the ESM and that may not have appeared as leading company at national level
Top three most sold packaged food and non-alcoholic beverage categories per country	Product category specific sales data per country	The different (or similar) most sold, and as such potentially most consumed, product categories per country
Most sold European packaged food and non-alcoholic beverage categories	Sum of the sales data per member state by year and product category	The different (or similar) most sold, and as such potentially most consumed, product categories across the ESM that may not have appeared among the top three at national level
Number of quick-service restaurant outlets per country	The number of outlets per 1000 inhabitants as obtained from Euromonitor	The different (or similar) density of quick-service restaurant outlets across the ESM
Number of annual fast food transactions per country	The number of transactions per 1000 inhabitants per year as obtained from Euromonitor	The different (or similar) amount of fast food transactions, and as such potential consumption levels, across the ESM
Dominant type of quick-service restaurant per country (chained versus independent)	The percent sales derived from chained consumer foodservice	The amount of fast food sales that can be attributed to larger quick-service restaurant chains
Preferred way of ordering and eating fast food per country	The percent of sales derived from eat in, take away, home delivery and drive through	The different (or similar) ways people across the ESM prefer to consume fast food
Number of supermarket outlets per country	Number of outlets per 1000 inhabitants as obtained from Euromonitor	The different (or similar) density of supermarket outlets across the ESM
Contribution of supermarket own-brand packaged food products to the overall sales of packaged foods per country	The percentage of packaged foods per country derived from supermarket own-brand products	The availability of supermarket own-brand packaged food products within the market per country. An estimation whether the sales of supermarket own-brand products is country specific
Contribution of supermarket own-brand non-alcoholic beverages to the overall sales of non-alcoholic beverages per country	The percentage of non-alcoholic beverages per country derived from supermarket own-brand products	The availability of supermarket own-brand non-alcoholic beverages within the market per country. An estimation whether the sales of supermarket own-brand products is country specific
**Market concentration**		
Herfindahl-Hirschman Index (HHI) per country	The summation of the squared market share of the firms active within the market and country	< 1000: Unconcentrated Markets; 1000–2000: Moderately Concentrated Markets; > 2000: Highly Concentrated Markets;
Four firm concentration ratio (CR4) per country	The combined market share of the four biggest firms active in the market and country	0: Perfect competition; 0–40: Effective Competition; 40–60: Limited competition; > 60: Dominant Firms with limited competition;

Data were first analysed by country and industry to obtain an overview of the market similarities and differences throughout the ESM. To compare market structure between food industries and across member states, analyses were conducted to identify the leading companies, and their respective market share for both national and global brand owners. In addition to the leading companies, the number of global brand owners with ≥1% market share and the number of unique companies per country and per food industry were identified to assess potential differences across countries. Unique companies were defined as companies having presence in only one ESM member state. The higher the number of global brand owners with ≥1% market share and the higher the number of unique companies, the more diverse the actors active within the respective food industry were assumed to be.

Additionally, data were pooled to obtain the total sales per global brand owner and product category across the ESM and as such to identify companies that may not have appeared as a leading company at national level, but overall hold a substantial market share at the European level. This was done by adding up the actual retail values per member state by year, by product category and by global brand owner.

Other analyses were conducted specific for different food industries. For packaged foods and non-alcoholic beverages, including own-brand products sold by supermarkets, the top three most sold product categories per country were identified based on retail sales value to understand whether these are similar throughout the ESM. For packaged foods, 14 product categories were included based on Euromonitor’s food categorization system, namely: ‘Ready meals’; ‘Sauces’, ‘Dressings and condiments’; ‘Soup’; ‘Sweet spreads’; ‘Dairy’; ‘Confectionery’; ‘Ice cream and frozen desserts’; ‘Savoury snacks’; ‘Sweet biscuits’, ‘Snack bars and fruit snacks’; ‘Baked goods’; ‘Breakfast cereals’; ‘Processed fruit and vegetables’; ‘Processed meat and seafood’; and ‘Rice, pasta and noodles’. For non-alcoholic beverages, eight different product categories were included, namely: ‘Carbonates’; ‘Concentrates’; ‘Juice’; ‘Ready-to-Drink Coffee’; ‘Ready-to-Drink Tea’; ‘Energy drinks’; ‘Sports drinks’ and ‘Asian speciality drinks’. The most sold product categories by retail sales value at the country level were in turn compared with the pooled data at the European level. The contribution of each product category to the total European sales of packaged foods and non-alcoholic beverages was calculated.

For both quick-service restaurants and supermarkets the number of outlets per 1000 inhabitants was obtained and compared between member states. Specifically, for quick-service restaurants, data pertaining to the dominant type of quick-service restaurant (i.e. chained versus independent), the amount of annual fast food transactions per 1000 inhabitants and the preferred way of ordering and eating fast food (i.e. eat in, take away, home delivery, drive through) per country were retrieved. Lastly, for supermarkets, the contribution of supermarket own-brand products to the overall sales of packaged foods and non-alcoholic beverages was examined for each country.

To assess levels of market concentration, the Herfindahl-Hirschman Index (HHI) and the four firm concentration ratio (CR4) were calculated. This was done by country for specific product markets within the packaged food and non-alcoholic beverage industries and for quick-service restaurants and supermarkets. Product markets were selected using Euromonitor’s food categorization system, as described above [23]. The HHI (calculated by summing the squared market shares) takes into account the market share of all players (with ≥1% market share) in the market. In comparison, the CR4 considers the combined market share of the four biggest firms active in the market. For the HHI the cut-off values as defined by the European Union (EU) merger regulations in 2004 (2004/C 31/03) were applied, with HHI-values below 1000 indicating unconcentrated markets and HHI-values above 2000 indicating concentrated markets [24]. CR4 values below 40% were in turn considered to represent a competitive market, values between 40 and 60% a market with limited competition and values above 60% were considered to indicate markets with limited competition and dominant firms in place [25]. An overview of the interpretation of the market concentration indices is given in Table 2.

In addition to the latest concentration indices in 2018 (2017 for quick service restaurants), the percent change of the HHI and the CR4 were calculated over the past 10 years (since 2009 for packaged food and non-alcoholic beverage manufacturers and since 2008 for quick service restaurants).

## Results

### Packaged food manufacturers

The top three most sold product categories in every member state of the ESM comprised of at least two of the three following product categories: ‘Dairy’, ‘Baked goods’, and ‘Processed meat and seafood’, contributing respectively 24, 18 and 15% to the overall European sales of packaged foods. ‘Dairy’ ranked as the most sold product category in 81% of the member states and ‘Baked goods’ in the five remaining member states (19%). In 37% of the member states, ‘Confectionery’ also entered the top three most sold product categories. This matched the fact that, according to the pooled ESM sales data, ‘Confectionery’ was the fourth most sold product category in Europe contributing 10% to the overall sales of packaged foods (data not shown).

Throughout the 27 ESM member states, 22 different global brand owner leader companies were identified with Mondelez International, Lactalis and Arla Foods Amba being the most reoccurring leading companies at the country level (Table 3). According to the pooled sales data throughout the ESM, Unilever Group and PepsiCo joined the list of aforementioned market leaders among the packaged food industry, although not being a leader producer of packaged food in any of the individual ESM member states. Shifting attention towards the national brand owners, in 13 out of the 27 ESM member states (48%), supermarkets were the leading brand owners through own-brand packaged food products placed on the market (data not shown).

**Table 3:**
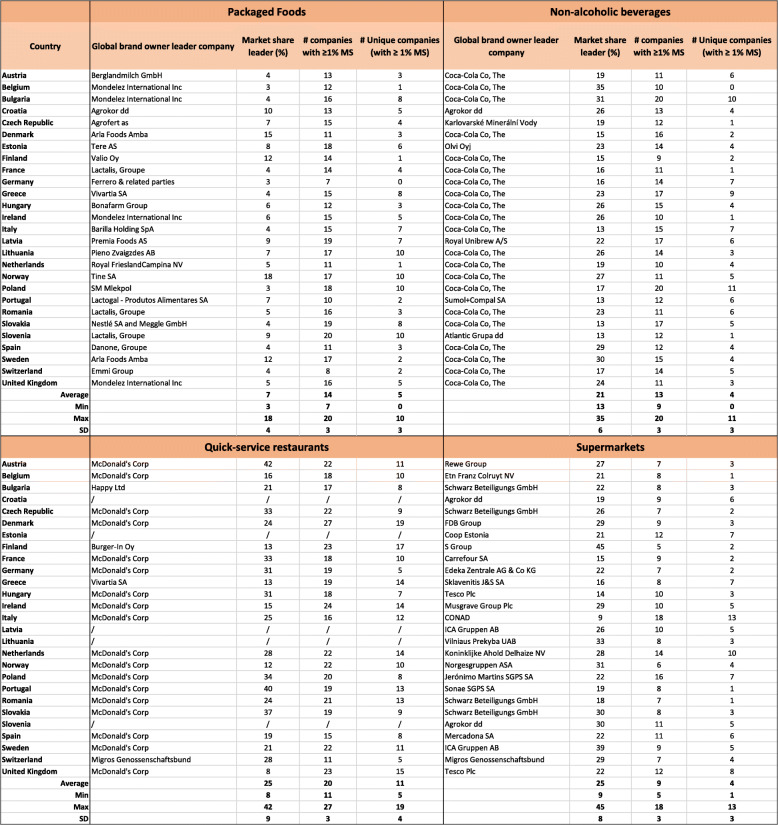
Global brand owner leading companies, the market share of the respective global brand owner leading companies (‘*Market Share leader (%)*’), the number of global brand owners with ≥1% market share (‘# *companies with ≥1% MS’*) and the number of unique companies (‘*# unique companies (with ≥1%MS)*’) per European Single Market (ESM) member state and food industry. Euromonitor data 2017/18

*MS* Market share

Assessing levels of market concentration, the product markets ‘Soup’, ‘Ice cream and frozen desserts’ and ‘Breakfast cereals’ were most concentrated, with an average CR4 across ESM member states of 72, 67 and 59%, respectively (Table 4). The CR4 for these three product markets was not lower than 40% in any ESM member state except for ‘Ice cream and frozen desserts’ in Italy (23%) and ‘Breakfast cereals’ in Finland (39%). The average CR4 across ESM member states amounted to around 40% or above for all 14 packaged food product markets, except for ‘Baked goods’ (21%), indicating limited competition. Similar levels of concentration were observed for the HHI (Appendix 1). The average concentration (both CR4 and HHI) for the packaged food industry slightly decreased between 2009 and 2018.

**Table 4:**
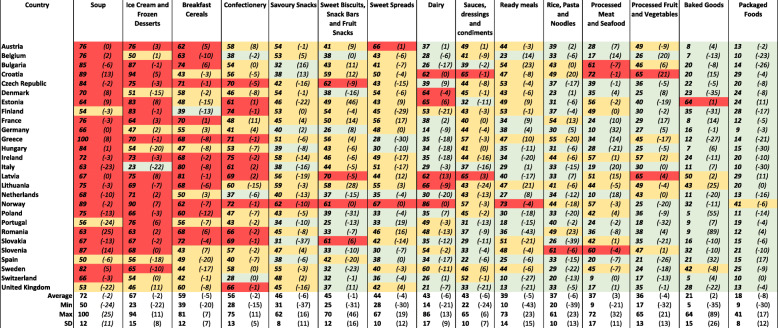
The four firm concentration ratio (CR4) for the 14 different packaged food product markets per European Single Market member state. Red indicates CR4 values > 60% and markets with dominant firms and limited competition, yellow indicates CR4 values between 40 and 60% and markets with limited competition while green indicates CR4 values ≤40% and markets with effective competition. The percent change over the past 10 years (2009–2018) is included in brackets. Euromonitor data 2018

### Non-alcoholic beverage manufacturers

The top three most sold non-alcoholic beverage product categories across ESM member states comprised ‘Carbonates’, ‘Juices’ and ‘Energy drinks’, contributing to 44, 30 and 11% of the overall European sales of non-alcoholic beverages, respectively. ‘Carbonates’ was the most sold product category in 89% of the ESM member states. Other product categories entering the top three were ‘Ready-to-Drink Tea’ and ‘Concentrates’, respectively in 19 and 11% of the ESM member states and contributing 6 and 5% to overall European non-alcoholic beverage sales (data not shown).

Throughout the 27 ESM member states, seven global brand owners were identified as being national market leaders. The Coca-Cola Company was the leading global brand owner in 21 of the member states (Table 3). Only in Croatia (Agrokor), the Czech Republic (Karlovarské Minerální Vody), Estonia (Olvi Oyj), Latvia (Royal Unibrew), Portugal (Sumol+Compal) and Slovenia (Atlantic Grupa) other leading global brand owners were observed. Where The Coca-Cola Company was not the leading company, they held the second largest market share in all countries except Slovenia. When looking at the pooled sales data throughout the ESM, additional market leaders within the non-alcoholic beverage industry were identified (PepsiCo, Nestlé, Danone and Suntory Holdings, data not shown).

According to the CR4 and HHI, the markets for ‘Carbonates’ and ‘Energy drinks’ were highly concentrated in most ESM member states. For both product markets the CR4 was on average 76% (Table 5). The HHI was 3069 and 2494, respectively (Appendix 2). The markets for ‘Ready-to-Drink Coffee’ and ‘Sport drinks’ joined the list with an average CR4 of 76 and 74% and an average HHI of 2852 and 2755, respectively. For the eight different non-alcoholic beverage product markets, the average CR4 did not go below 52%. Germany was the only country in which the CR4 was lower than 40% for all product markets except for ‘Carbonates’, ‘Ready-to-drink Tea’ and ‘Energy drinks’. The average HHI did not go below 2000 for any non-alcoholic beverage product market except for ‘Concentrates’ and ‘Juices’ (Appendix 2). In contrast to the packaged food markets, the concentration of the non-alcoholic beverage markets increased from 2009 to 2018 according to both the average CR4 and the HHI. Summarized, the CR4 and HHI indicated moderately to highly concentrated markets (Table 5).

**Table 5:**
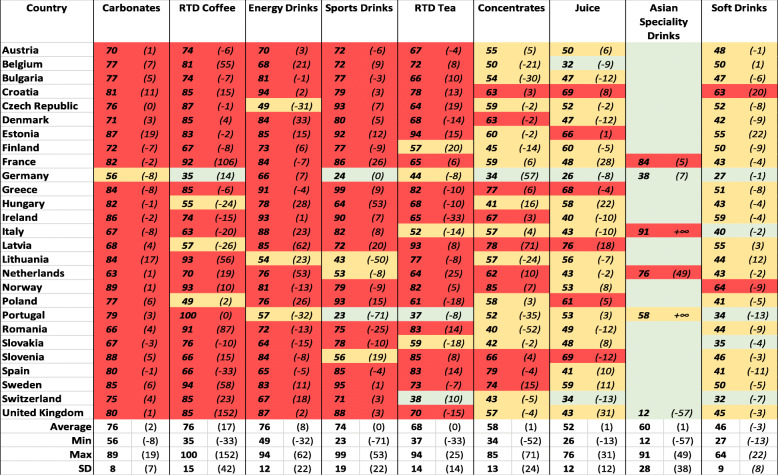
The four firm concentration ratio (CR4) for the 8 different non-alcoholic beverage product markets per European Single Market member state. Red indicates CR4 values > 60% and markets with dominant firms and limited competition, yellow indicates CR4 values between 40 and 60% and markets with limited competition while green indicates CR4 values ≤40% and markets with effective competition. Between brackets the percent change over the past 10 years is included (2009–2018). Euromonitor data 2018

*RTD* Ready-to-drink. For ‘Asian Specialty Drinks’ data were lacking in several countries

### Quick-service restaurants

Within the ESM in 2017, on average across member states, 20% of the quick-service restaurant sales came from international chains or restaurants with 10 or more outlets in the country (with a minimum of 7% in Italy and going up to 44% in the United Kingdom, data not shown). Consumers spent more on eat-in than take-away, home-delivery and drive-through. On average 77% (min 64% in France to max 86% in Austria) of the sales could be attributed to meals consumed in the restaurant. Drive-through seemed to be the least popular in the ESM, only contributing on average 1% to the sales (min 0% in Greece up to max 3% in France). Take-away and home-delivery on average contributed 16 and 5%, respectively (data not shown). Per 1000 inhabitants, a country within the ESM in 2017 on average counted 3.7 quick-service restaurant outlets. The lowest number was observed in Romania (1.3 outlets/1000 inhabitants) and the highest in Portugal (8 outlets/1000 inhabitants). The annual average number of quick-service restaurant transactions within the ESM in 2017 was 91,651 per 1000 inhabitants (46,499 in Poland up to 217,372 in Spain, per 1000 inhabitants) (data not shown).

In all 22 ESM member states for which data were available, except Greece, McDonald’s was the leading company (82%) or the company with the second largest market share (14%) with on average 1.8 outlets per 1000 inhabitants (with a minimum of 0.2 in Greece and a maximum of 4.2 in Switzerland, data not shown). Other companies that held the leader position were Happy Ltd. in Bulgaria, Burger-In Oy in Finland, Vivartia in Greece and Migros Genossenschaftsbund in Switzerland (Table 3).

The CR4 was 50% on average and did not go below 40% in any of the ESM member states apart from Ireland and the United Kingdom (Table 6). In contrast, the HHI indicated unconcentrated markets in 50% of the ESM member states. This discrepancy was also observed when looking at the percent change from 2008 to 2017. While the CR4 had increased in all the ESM member states, the HHI had decreased in all except Austria, the Netherlands, Poland and Spain. This difference between both concentration indices could be attributed to the market share of the top four firms increasing as well as being more evenly distributed.

**Table 6:**
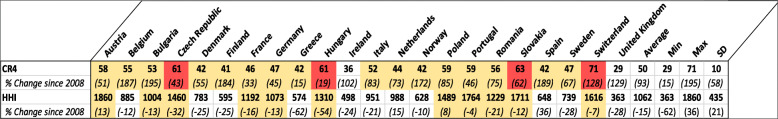
The four firm concentration ratio (CR4) and the Herfindahl-Hirschman Index (HHI) for the quick-service restaurant industry per European Single Market member state. Red indicates CR4 values > 60% and HHI values > 2000 so highly concentrated markets, yellow indicates CR4 values between 40 and 60% and HHI values between 1000 and 2000 so moderately concentrated markets and green indicates CR4 values ≤40 and HHI < 1000 so unconcentrated markets. Between brackets the percent change over the past 10 years is included (2008–2017). Euromonitor data 2017

### Supermarkets

For the purpose of this analysis, supermarkets were considered as manufacturers of packaged foods and non-alcoholic beverages through own-brand products placed on the market and as retailers selling the products. Among the packaged foods and non-alcoholic beverages available on the market, 15% (SD = 8.8) of the packaged foods and 7% (SD = 5.5) of the non-alcoholic beverages could be attributed to supermarket own-brand products. Within Estonia no supermarket had a market share of ≥1% for selling own-brand packaged food products. In contrast, in Switzerland, 39% of the sold packaged food products were supermarket own-brand products. For the sales of non-alcoholic beverages a similar picture could be observed as for packaged foods. In Romania and Greece no supermarket had a market share of ≥1% for selling own-brand non-alcoholic beverages. In Switzerland, 23% of the non-alcoholic beverage sales were supermarket own-brand products. This suggested that the role of supermarkets as producers of own-brand packaged foods and non-alcoholic beverages was country specific. A country within the ESM on average counted 2.4 supermarket outlets per 1000 inhabitants. This figure amounted to one outlet per 1000 inhabitants in nine ESM member states (Austria, Denmark, Finland, Germany, Ireland, Norway, Slovenia, Sweden and the United Kingdom) and up to six in Bulgaria and Greece (in 2018, data not shown).

The most reoccurring supermarket within the ESM was Schwarz Beteiligungs (*brands: Kaufland, Lidl and Plus*) being the global brand owner in four countries and having a presence in 24 of the 27 ESM member states. Other supermarkets playing a leading role in several countries were Agrokor (*brands: Getro, Hura!, Konzum, Mercator, Slobodna Dalmacija and Tisak*) Tesco (*brands: One Stop, S-Market, Savia, Tesco and Zabka*) and ICA Gruppen (*brands: ICA, Rimi, Supernetto and Säästumarket*), all being the leader in two ESM member states and having a presence in two, six and four member states, respectively.

Although several different supermarkets were present throughout the ESM, noteworthy concentration took place at national level with an average CR4 of 60% (Table 7). The CR4 only dropped below 40% in Bulgaria, Greece, Italy and Romania and did not go below 30% in any of the ESM member states. The average HHI within the ESM member states stood at 1245 with highly concentrated markets (> 2000) in Finland, Norway and Sweden. In 44% of the ESM member states the HHI remained below 1000 indicating unconcentrated markets. Within these unconcentrated markets however, only 33% of the member states also had a CR4 below 40%. In 82% of the ESM member states both the CR4 and HHI had increased since 2009 (Table 7).

**Table 7:**
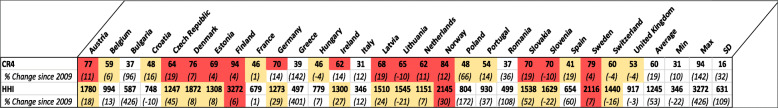
The four firm concentration ratio (CR4) and the Herfindahl-Hirschman Index (HHI) for the supermarket industry per European Single Market member state. Red indicates CR4 values > 60% and HHI values > 2000 so highly concentrated markets, yellow indicates CR4 values between 40 and 60% and HHI values between 1000 and 2000 so moderately concentrated markets and green indicates CR4 values ≤40 and HHI < 1000 so unconcentrated markets. Between brackets the percent change over the past 10 years is included (2008–2017). Euromonitor data 2017

Summarized, it was concluded that, even though the overall market remained relatively unconcentrated in most ESM member states, most of the market share tended to be controlled by the four biggest national supermarkets.

### Combined results for the four food industries

**Fig. 1:**
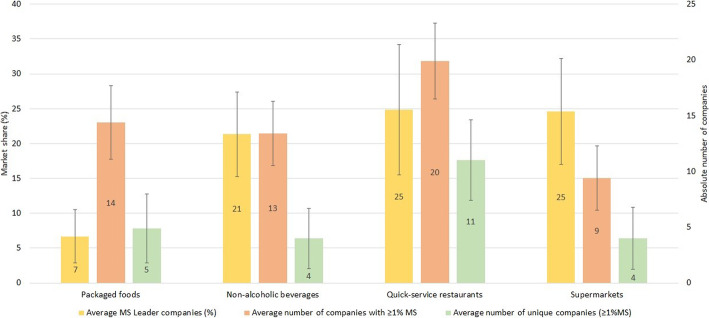
Average market share (MS) in hands of the leading global brand owner company (yellow), average number of global brand owners with ≥1% market share (orange) and average number of unique companies (green) across European Single Market member states and per food industry. The error bars indicate the respective standard deviation

As shown in Fig. 1, both the average number of global brand owners per country with ≥1% market share and unique companies per country with ≥1% market share across ESM member states tended to be lower among supermarkets than what was observed for packaged food and non-alcoholic beverage manufacturers and quick-service restaurants. A ESM member state on average counted 14 packaged food global brand owners with ≥1% market share (minimum 7 in Germany up to maximum 20 in Slovenia), 13 non-alcoholic beverage companies (minimum 9 in Finland up to 20 in Bulgaria and Poland), 20 quick-service restaurants (minimum 11 in Switzerland and 27 in Denmark) and nine supermarkets (minimum five in Finland and maximum 18 in Italy). Similar results were observed for the unique companies, with a ESM member state on average having five unique packaged food companies (no unique companies in Germany going up to ten in Lithuania, Norway, Poland and Slovenia), four unique non-alcoholic beverage companies (no unique companies in Belgium to maximum 11 in Poland), 11 unique quick-service restaurants (minimum five in Switzerland and Germany going up to 19 in Denmark) and four unique supermarkets (minimum one in Belgium, Portugal and Romania and maximum 13 in Italy) (Table 3, Fig. 1).

In contrast, the average market share per country in the hands of the leading global brand owners was the highest for quick-service restaurants and supermarkets, with both holding, on average per country, 25% market share (minimum 8% to maximum 42% for quick-service restaurants and minimum 9% to maximum 45% for supermarkets). The average market share per country in the hands of the leading packaged food and non-alcoholic beverage company was 7% (3–18%) and 21% (13–35%), respectively (Table 3, Fig. 1).

The considerably higher average number of global brand owners with ≥1% market share and unique companies per country among quick-service restaurants was indicative of a higher in-country diversity of quick-service restaurants. This was not observed for supermarkets.

## Discussion

Using Euromonitor sales and market share data, this study set out to provide an analysis of the food industry within the ESM, comparing aspects of market structure for four food industries, namely packaged foods, non-alcoholic beverages, quick-service restaurants and supermarkets. Substantial differences were found across European countries and food industries. For packaged food and non-alcoholic beverage manufacturers similar companies and most sold product categories were observed throughout the ESM with the main difference between both industries being the higher level of market concentration within the non-alcoholic beverage industry and respective product markets. For quick-service restaurants the same leading companies were detected throughout Europe with increased market share moving towards the four largest companies since 2008. In spite of these levels of market concentration, quick service restaurants showed to have a considerable higher number of global brand owners with ≥1% market share and unique companies than any other food industry. In contrast, supermarkets were shown to have a diversity of companies throughout Europe, but noteworthy concentration took place at country level with most of the market share being in hands of the four national supermarkets with the largest market share. This was also reflected in the lower number of global brand owners with ≥1% market share and unique companies.

Our data showed that the most sold packaged food and non-alcoholic beverage categories were similar throughout Europe with ‘Baked goods’, ‘Dairy’, ‘Processed meat and seafood’ and ‘Confectionery’, contributing a combined 67% to the overall European sales of packaged foods and ‘Carbonates’, ‘Juices’ and ‘Energy drinks’ contributing to 85% of the sales of non-alcoholic beverages. The companies selling these product categories were also similar across Europe with a country on average having only five unique packaged food companies and four non-alcoholic beverage companies.

These similar players and most sold product categories across the ESM suggest that from a public health point of view the market for packaged foods and non-alcoholic beverages could be approached as one territory and could facilitate the implementation of regulations affecting packaged food and non-alcoholic beverage manufacturers at a European level. Implementing regulations such as marketing restrictions (for certain media like food packages, internet and social media), reformulation targets and front-of-pack labelling at a European level would potentially be preferable to pursuing national policy measures from a public health point of view. This would ensure policy consistency across the region and would be likely to ease the administrative burden associated with policy development and implementation. Furthermore, a harmonised policy framework across the ESM would likely facilitate implementation from a food industry point of view, as has been argued by some companies that have pushed for the Nutri-Score to be made mandatory at European level [26–28]. For the moment a variety of policy measures are already in place throughout the ESM, but the policy content and implementation varies by country [3, 29]. The trans-fat regulation and obligatory on-pack nutritional information (detailing how much energy and nutrients a product contains) are examples of successful European-wide legislation in this area [30, 31].

Our data showed that in about 50% of the ESM member states, supermarkets were the leading national brand owners selling packaged foods through own-brand products placed on the market. However, their role as producers of packaged foods and non-alcoholic beverages varied significantly throughout the ESM. In addition, in most ESM member states, the combined market share of the four biggest supermarkets was on average 60% (31–94%). This places them in a unique position for in-store health promoting interventions with the potential to influence purchasing behaviour of a significant proportion of the population. Currently only limited voluntary initiatives have been made by supermarkets in the Netherlands, the United Kingdom and Austria introducing healthy checkout counters [32–36]. Studies have, however, shown that all-inclusive interventions combining price incentives, nutritional information and easy access to healthy foods could considerably help improve the in-store food environment [37]. Nonetheless, our data showed that the role of supermarkets is rather country specific and as such regulations affecting the in-store environment would potentially benefit from being implemented at a national level. However, first more research is needed to summarize the commitments already made by supermarkets and identify policy options adapted to the national food environment that could help ensure that supermarkets use their unique position to move the market in a healthier direction.

Alongside supermarkets, quick-service restaurants have an important role within the food environment [38–40]. Our results showed that ESM member states on average have more quick-service restaurant outlets than supermarkets (3.7 and 2.4 per 1000 inhabitants, respectively). Although, among quick-service restaurants on average 50% of all the market share was in hands of the four biggest companies, the industry also counted the highest average number of unique companies for one ESM member state and companies with ≥1% market share compared to packaged food manufacturers, non-alcoholic beverage manufacturers and supermarkets. The latter was reflected in the low concentration levels according to the HHI. These data suggest that, even though the bigger players are present in most of the ESM member states, smaller players at national level are important and should be taken into account when formulating nutrition policies. As such, similar to supermarkets, regulations affecting quick-service restaurants could potentially benefit from being implemented at national level. Potential policies could be the implementation of nudging techniques and menu-labelling which have shown to be effective in schools and among non-overweight individuals, respectively [38, 39, 41, 42]. However, first more research is required to identify the unique national companies, understand the national food environment and summarize the commitments already made by quick-service restaurants.

Within the abovementioned four food industries and respective product markets, our data indicated moderately to highly concentrated markets. These levels of market concentration may be of concern from a public health perspective for a number of reasons, including how the extra profits may be used to support or hamper the implementation of government policies affecting the food environment [16, 17]. This is especially of concern when many of the product portfolios of the companies consist of less healthy products. Selling less healthy, but more profitable products in concentrated markets can in turn increase profit margins [43, 44]. These profits can then be used to fund corporate practices, such as marketing of unhealthy food, lobbying and paying fees to supermarkets to place unhealthy products at favourable locations in the shop, that may undermine public health efforts to improve population diets [16, 43]. However, to understand to what extent such practices take place, more research into European and country specific corporate activities is required.

The study has several strengths. Most importantly, this study forms a basis to understand how certain aspects of the market structure of key European food industries may influence food environments. A key strength of the study is the amount of data used to identify the similarities and differences across Europe as well as the levels of concentration per food industry and respective product markets. It also highlights the importance of a transdisciplinary approach, not only taking into account the effectiveness of policies to improve the food environment, but additionally looking at the economic environment surrounding it. There were however also limitations identified. The Euromonitor database is based around the ownership of brands (e.g. national and global brand owners) rather than companies. As a result, the global brand owners identified may change when brands are sold to new brand owners. Further, having looked at the aforementioned levels of concentration it must be kept in mind that these may be an underestimation. Companies being considered independent in Euromonitor (due to the database being built around brand ownership), and as such for the concentration calculations, may still sell well-known brands from other companies through licensing agreements. In addition, not all products within one food category, as determined by the Euromonitor’s food categorization system, are interchangeable from a consumer point of view (for example, the category ‘Baked goods’ contains both bread and pastries). Hence, levels of concentration may increase when calculating the concentration indices for more specific food categories. Furthermore, for this study the geographic boundaries were defined based on the available data (at national level using Euromonitor’s food categorisation system), but in reality, the geographic boundaries, especially for supermarkets and quick-service restaurants, may be different to national boundaries [23, 45, 46]. In addition, to further assess market structure, other aspects should be considered, such as barriers to entry and degree of vertical integration. Another step towards the future is to connect the players with the largest market share per food industry with their nutritional commitments and the healthiness of their product portfolios to identify gaps between commitments and performance and point out areas that could be improved by the implementation of nutrition policies.

## Conclusions

This study provided an analysis of the packaged food manufacturing, non-alcoholic beverage manufacturing, quick service restaurant and supermarket industries within the ESM. While similarities in market structure throughout the ESM were observed for packaged food and non-alcoholic beverage manufacturers, a different picture was seen for quick-service restaurants and supermarkets. The first displayed a remarkably higher diversity of companies at the national level while the latter demonstrated the contrary. Due to these structural differences between food industries, a differentiation between European and national level regulations by industry was suggested to potentially facilitate the implementation of nutrition policies. This study highlights the importance of a transdisciplinary approach taking into account not only the effectiveness of nutrition policies to improve the food environment, but also the economic environment surrounding it.

## Figures and Tables

**Table 8:**
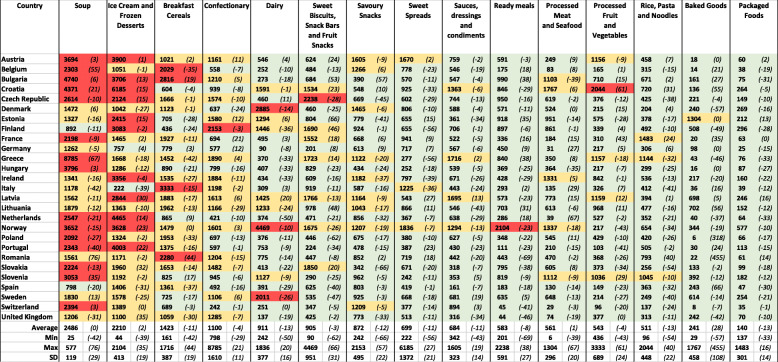
The Herfindahl-Hirschman Index (HHI) for the 14 different packaged food product markets per European Single market member state. Red indicates HHI values > 2000 and highly concentrated markets, yellow indicates HHI-values between 1000 and 2000 and moderately concentrated markets and green indicates HHI-values < 1000 and unconcentrated markets. Between brackets the percent change over the past 10 years is included (2009–2018). Euromonitor data 2018

**Table 9:**
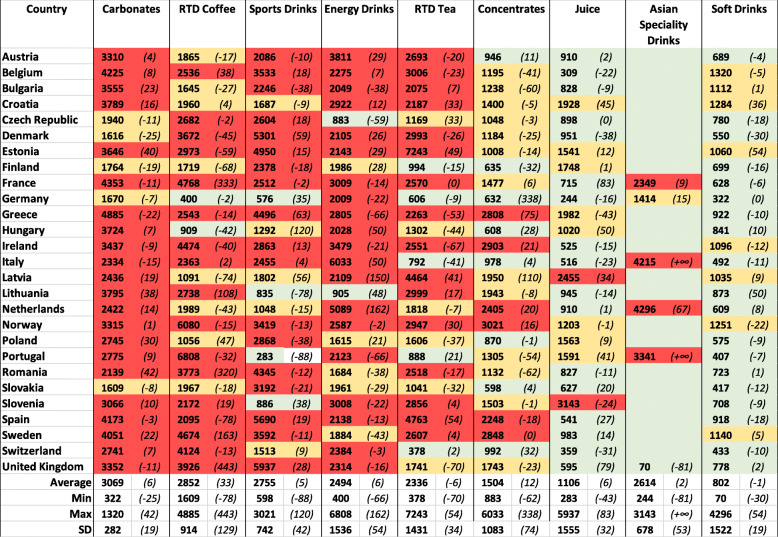
The Herfindahl-Hirschman Index (HHI) for the 8 different non-alcoholic beverage product markets per European Single Market member state. Red indicates HHI values > 2000 and highly concentrated markets, yellow indicates HHI-values between 1000 and 2000 and moderately concentrated markets and green indicates HHI-values < 1000 and unconcentrated markets. Between brackets the percent change over the past 10 years is included (2009–2018). Euromonitor data 2018

*RTD* Ready-to-drink. For ‘Asian Specialty Drinks’ data were lacking in several countries

## Data Availability

The datasets used and/or analysed during the current study are available from the corresponding author on reasonable request.

## Abbreviations

BMI: Body Mass Index
CR4: Four firm concentration ratio
ESM: European Single Market
GBO: Global Brand Owner
HHI: Herfindahl-Hirschman Index
NBO: National Brand Owner
